# Feature engineering and parameter tuning: improving phenomic prediction ability in multi-environmental durum wheat breeding trials

**DOI:** 10.1007/s00122-024-04695-w

**Published:** 2024-07-22

**Authors:** Carina Meyenberg, Vincent Braun, Carl Friedrich Horst Longin, Patrick Thorwarth

**Affiliations:** State Plant Breeding Institute, University of Hohenheim, Fruwirthstr. 21, 70599 Stuttgart, Germany

## Abstract

**Key Message:**

Optimized phenomic selection in durum wheat uses near-infrared spectra, feature engineering and parameter tuning. Our study reports improvements in predictive ability and emphasizes customized preprocessing for different traits and models.

**Abstract:**

The success of plant breeding programs depends on efficient selection decisions. Phenomic selection has been proposed as a tool to predict phenotype performance based on near-infrared spectra (NIRS) to support selection decisions. In this study, we test the performance of phenomic selection in multi-environmental trials from our durum wheat breeding program for three breeding scenarios and use feature engineering as well as parameter tuning to improve the phenomic prediction ability. In addition, we investigate the influence of genotype and environment on the phenomic prediction ability for agronomic and quality traits. Preprocessing, based on a grid search over the Savitzky–Golay filter parameters based on 756,000 genotype best linear unbiased estimate (BLUE) computations, improved the phenomic prediction ability by up to 1500% (0.02–0.3). Furthermore, we show that preprocessing should be optimized depending on the dataset, trait, and model used for prediction. The phenomic prediction scenarios in our durum breeding program resulted in low-to-moderate prediction abilities with the highest and most stable prediction results when predicting new genotypes in the same environment as used for model training. This is consistent with the finding that NIRS capture both the genotype and genotype-by-environment $(G\times E)$ interaction variance.

**Supplementary Information:**

The online version contains supplementary material available at 10.1007/s00122-024-04695-w.

## Introduction

Plant breeding aims to develop superior cultivars by selecting the best individuals. Since very little phenotypic information is available in early generations, breeders aim to predict the genetic value in order to select the best individuals already early in the breeding cycle. In addition to genomics, based on molecular marker data (Meuwissen et al. 2001), other omics data such as transcriptomics (Westhues et al. 2017) or metabolomics (Schrag et al. 2018; Thorwarth et al. 2019) have been used to predict the phenotypic performance. Despite reductions in application cost, these methods are often still too expensive for a large-scale routine use in breeding programs.

In 2004, Ferrio et al. used near-infrared spectra (NIRS) obtained on durum wheat flour to predict grain yield (GY) using partial least squares regression (PLSR). Based on their results, they concluded that an accurate estimation of GY was not possible. However, Rincent et al. (2018) proposed to use NIRS in combination with models classically used for genomic selection and called the method phenomic selection. Based on their findings in wheat and poplar, they reported phenomic selection to be as good as genomic selection, which motivated further studies to investigate its potential in further crops (Brault et al. 2022; Lane et al. 2020; Robert et al. 2022a, 2022b, 2022c; Weiß et al. 2022; Zhu et al. 2022, 2021; Dallinger et al. 2023). All of these studies have in common, that the NIRS were preprocessed before being used for phenomic prediction. Spectra preprocessing is a fundamental part of working with spectral data (Rinnan et al. 2009). Different preprocessing techniques exist, but they all have in common to reduce unwanted spectral noise, to enhance the signal by highlighting absorption features in the spectral data and to resolve overlapping signals (Delwiche and Reeves 2010; Barak 1995; Rinnan et al. 2009; Savitzky and Golay 1964; Zimmermann and Kohler 2013). In the aforementioned studies on phenomic prediction, NIRS were always preprocessed with the Savitzky–Golay filter (Savitzky and Golay 1964) before using the spectral data for prediction. The Savitzky–Golay filter is a data smoothing method based on a local least squares polynomial regression inside a moving window, in which the neighboring points are used to determine the best polynomial fit and consequently smooth the data (Barak 1995; Delwiche and Reeves 2010; Zimmermann and Kohler 2013). When applying the Savitzky–Golay filter to spectral data, three parameters must be defined, namely the derivative order, the polynomial order and the window size. As studies from chemometric modeling show, it may be worthwhile to tune the Savitzky–Golay filter parameters to obtain better predictions (Delwiche and Reeves 2010), which to the best of our knowledge has not been analyzed in detail in phenomic selection for plant breeding programs.

In spectroscopy-based studies e.g., from cereal sciences, the arithmetic mean for each genotype is calculated (Rincent et al. 2018) or individual samples within each genotype are often used for the prediction (Nagel-Held et al. 2023, Nagel-Held et al. 2022, Robert et al. 2022b). However, in plant breeding, adjusted means are usually calculated based on linear mixed models in the form of BLUEs or BLUPs (Piepho et al. 2008) to correct for the experimental design effects. Little research has been done to identify the best preprocessing of NIRS for the use in phenomic prediction. To the best of our knowledge, only Brault et al. (2022) have investigated the adjustment effects in the context of phenomic prediction, with comparing mean and BLUP calculations for NIRS and trait data resulting into higher prediction abilities when using BLUPs compared to the means. There are several methods for NIRS that can improve the prediction ability (Rinnan et al. 2009). A promising method from the field of machine learning is feature engineering: the process of selecting, transforming or creating features from raw data that improve the prediction ability (Duboue 2020). In addition to feature engineering and parameter tuning, the choice of the prediction model also affects the phenomic prediction ability (Lane et al. 2020; Zhu et al. 2021).

Durum wheat (*Triticum turgidum *ssp. durum Desf.) is one of the most important cereal species, grown today mainly for the pasta production, but also as the basis for couscous and bulgur (Beres et al. 2020). Durum wheat is an autogamous species, so classical line breeding methods such as pedigree, bulk or single-seed descent are used to improve, among other traits, grain yield and grain quality (Xynias et al. 2020). Phenomic prediction could be an addition to the breeder’s toolbox to accelerate the breeding progress in durum wheat, which was to the best of our knowledge not yet investigated with data of a real durum wheat breeding program. Therefore, we investigated the potential of NIRS for predicting phenotype performance using data from multi-environmental breeding programs and evaluated the application of phenomic prediction in different breeding scenarios. In addition, we performed feature engineering and Savitzky–Golay filter parameter tuning in order to improve the phenomic prediction ability and investigated the mechanisms underlying phenomic prediction.

## Materials and methods

Eight durum wheat datasets, namely Central European durum wheat panel (CP), South European durum wheat panel (SP), durum wheat yield trial 1 (YT1) conducted in 2019, 2021 and 2022 and durum wheat yield trial 2 (YT2) conducted in 2020, 2021 and 2022 were analyzed in this study. In all eight datasets, durum wheat was grown in a winter cropping system with fall sowing and harvest in the summer of the subsequent year.

### Plant material, field trials and phenotypic data

The CP is a diverse set of 189 durum wheat genotypes adapted to the more Central and Eastern European climate. It includes ancient varieties, modern varieties and breeding lines (Rapp et al. 2019; Sieber et al. 2017). The field experiments were conducted in the 2015/2016 growing season, and data from the three environments Aschersleben (ASL), Hohenheim (HOH) and Martonvásár (MAR) were included in the analysis (Fig. S1, Table S1). The CP set as previously described in Sieber et al. (2017) and Rapp et al. (2018) was subset to these three environments, due to the NIRS availability. Depending on the environment, the plots varied in size between 6 and 11 m^2^ and were laid out as a partially replicated design in each environment.

The SP consists of 159 durum wheat genotypes predominantly grown in France and adapted to a more Southern or Western European climate (Rapp et al. 2018). The SP was grown in the 2014/2015 growing season and data from six environments Eckartsweier (EWE), Hohenheim (HOH), L’Isle-Jourdain (ISL), Prunay-le-Gillon (PRU), Saint Jean d’Angély (JEA) and Reuilly (REU) were considered (Fig. S1, Table S1). The plots were laid out in a partially replicated design in each environment and varied in size between 6 and 11 m^2^, depending on the environment.

*YT1* corresponds to the first cycle yield evaluation of breeding lines of the winter durum wheat breeding program at the State Plant Breeding Institute of the University of Hohenheim tested in 2019, 2021 and 2022 at two environments each (Fig. S1, Table S1). It consists of relatively frost-resistant F_5_-lines. The plots were laid out in an $\alpha$-lattice design with two replications within one environment (2019 in HOH and EWE, 2021 in HOH), respectively, one replication within one environment (2021 in Rastatt (RAS), 2022 in HOH, EWE) and varied in size from 6 to 9 m^2^ depending on the environment.

*YT2* corresponds to the first cycle yield evaluation of breeding lines of the summer durum wheat breeding program at the State Plant Breeding Institute of the University of Hohenheim tested in 2020, 2021 and 2022 at two environments each (Fig. S1, Table S1). In contrast to YT1, YT2 consists of F_5_-lines rather susceptible to frost. The plots of YT2 were laid out in an $\alpha$-lattice design with two replications within one environment (2020 in EWE and HOH), respectively, one replication within one environment (2021 in HOH and RAS, 2022 in ASL and EWE) and varied in size from 6 to 11 m^2^ depending on the environment.

Field plots were harvested with a combine harvester, and grain yield (GY) was determined in t ha^−1^ and adjusted for moisture content. Protein content (PC) in % was determined on grains using a stationary NIR spectroscope (SpectraStar 2400 RTW, Unity Scientific, USA) at 22 °C room temperature and a custom NIRS calibration (International Association for Cereal Science and Technology (ICC) standard method 159, Vienna, Austria).

### NIRS data

Near-infrared spectra were obtained for all grain samples in each environment of each dataset. Therefore, all grain samples were dried to a uniform moisture content of 14%. At 22 °C room temperature, reflectance intensity was measured using a SpectraStar 2400 RTW spectrometer (Unity Scientific, Milford, MA, USA) over a range of 1300 nm–2300 nm in 1 nm increments. Each grain sample was measured three times with the spectrometer. The arithmetic mean (mean) of the three measurement replications was used as the final spectrum for the subsequent analysis. Please note that ‘mean’ as used below always refers to the arithmetic mean. The NIRS were exported using SL Data Manager software (Carl Zeiss Spectroscopy GmbH, Germany), and the subsequent NIRS preprocessing was performed using *R* software (R Core Team 2022) and the *R* package ‘prospectr’ (Stevens and Ramirez-Lopez 2022).

### Phenotypic and NIRS data analysis

Summary statistics including the minimum, mean and maximum as well as the heritability of the agronomic traits were determined. Due to the partially replicated experimental design and the corresponding unbalanced data, the broad-sense heritability was calculated as follows: ${H}^{2}=1-\frac{{\overline{\vartheta }}_{BLUP}}{2 {\sigma }_{g}^{2}}$, where $\overline{\vartheta }$ is the mean variance of a difference of two BLUPs (best linear unbiased predictor) and ${\sigma }_{g}^{2}$ the genotype variance (Cullis et al. 2006; Piepho and Möhring 2007; Schmidt et al. 2019). As the datasets of YT1 and YT2 contain data from breeding programs, where the environments represent the replications, both effects are confounded and the variance of the $G\times E$ -interaction cannot be estimated.

Moreover, linear mixed models were used to obtain best linear unbiased estimates (BLUEs) of the genotypes and to estimate variance components over each dataset series (CP, SP, CP and SP combined, YT1-2019, YT1-2021, YT1-2022 and YT2-2020, YT2-2021, YT2-2022). Dataset series means that data from all environments belonging to the corresponding dataset have been used. All linear mixed models were run using *R* software version 4.2.2 (R Core Team 2022) and the *R* package ‘ASReml-*R*’ version 4.1.0.1 (Butler et al. 2017). For the CP and SP datasets, which are connected by in total 20 genotypes, each wavelength was subjected to the same linear mixed model as the agronomic traits. For the estimation of genotype BLUEs, genotype was considered as fixed effect and all other effects were modeled as random, whereas for the estimation of variance components, all effects, including genotype, were modeled as random. The linear-mixed model had the following general parametrization:

1 $$y_{ijsh} = \mu + g_{i} + l_{j} + r_{js} + b_{jsh} + gl_{ij} + \in_{ijsh}$$

where ${y}_{ijsh}$ is the observed trait (agronomic trait or NIR wavelength) for each plot, $\mu$ the general mean, ${g}_{i}$ denotes the genotype effect, ${l}_{j}$ the environment effect, ${r}_{js}$ the random replication effect nested within the $j$th-environment, ${b}_{jsh}$ the random incomplete block effect nested within the $s$th-replication at the $j$th-environment, ${gl}_{ij}$ is the $G\times E$-interaction, and ${\epsilon }_{ijsh}$ the residual error. All random effects were assumed to be normally distributed with a mean of zero and own variance such that ${l}_{j}\sim N(0,{\sigma }_{l})$, ${r}_{js }\sim N(0,{\sigma }_{r})$, ${b}_{jsh }\sim N(0,{\sigma }_{b})$, ${gl}_{ij}\sim N(0,{\sigma }_{gl})$ and ${\epsilon }_{ijsh}\sim N(0,{\sigma }_{\epsilon })$.Variance component estimates were calculated using the restricted maximum likelihood (REML) approach (Cochran and Cox 1957). For the YT datasets, the genotype BLUEs of the series, as well as the genotype BLUEs within single environments with two replications per environment, were calculated similarly, but without the replication effect and the environment effect including its interaction terms. Given the situation, that for few YT datasets NIRS of just one replication per environment were available, the raw spectra were used instead of the BLUEs. In addition to that, if for some wavelengths, no BLUEs could be calculated for single environments, due to singularities, the affected wavelengths were subsequently removed for all environments used for the respective predictions.

### Feature engineering and parameter tuning

**Fig. 1:**
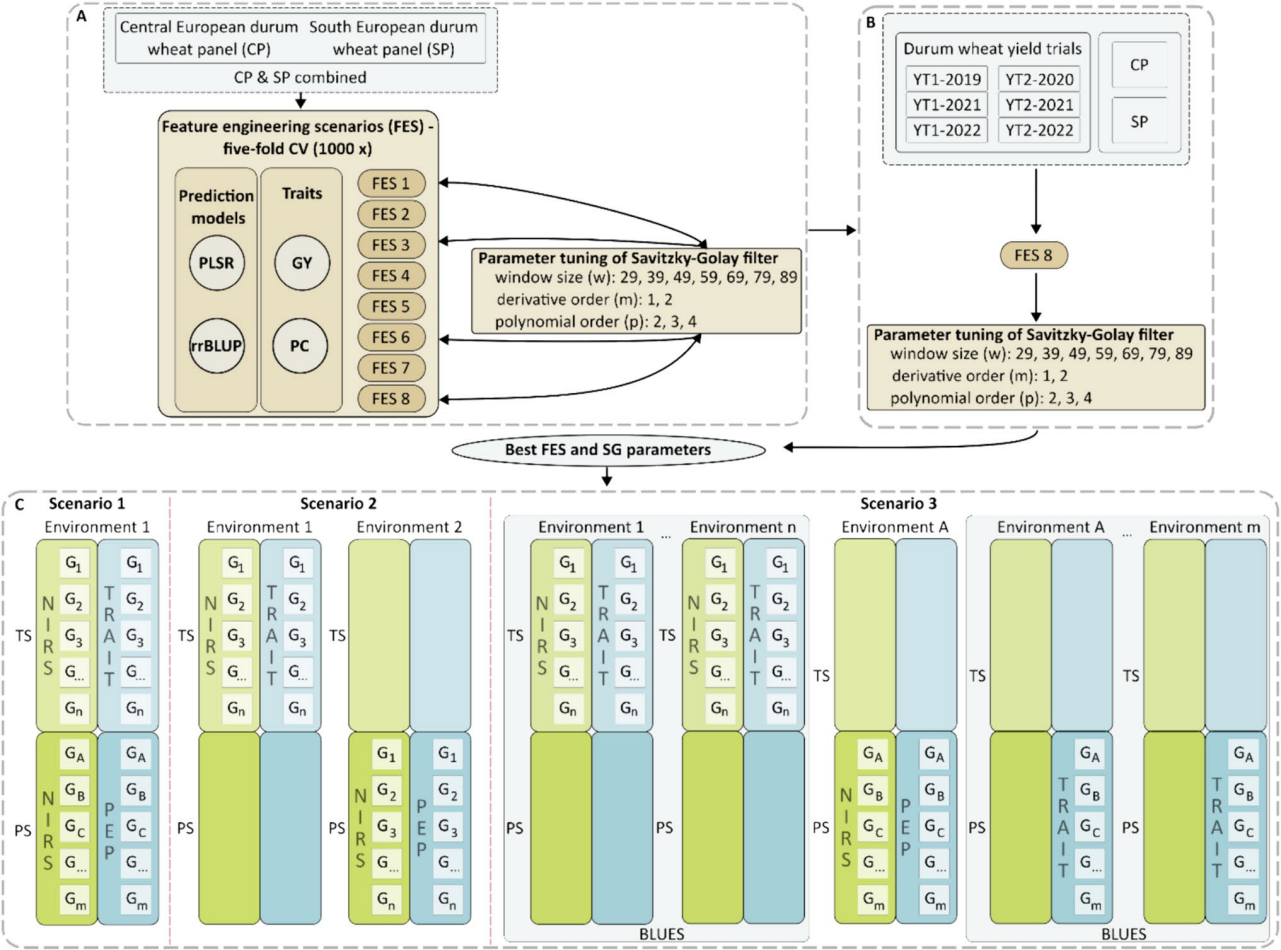
Schematic overview of NIRS and trait data preprocessing including the feature engineering scenarios (FESs, **A**) and the Savitzky–Golay filter parameter tuning (**B**). Moreover, the three breeding scenarios evaluated in this study are depicted (**C**). **A** For the dataset ‘CP & SP combined’ and the traits grain yield (GY) and protein content (PC), eight FESs, each tested with the two prediction models partial least squares regression (PLSR) and ridge regression best linear unbiased prediction (rrBLUP) were evaluated to identify the FES resulting in the highest cross-validated prediction ability within the tested range of parameters. Therefore, a fivefold cross validation, with random assignment of genotypes to folds, replicated 1000 times was conducted. Simultaneously, the Savitzky–Golay filter parameter tuning was conducted to obtain the highest Savitzky–Golay filter parameter combination within the tested range of parameters. **B** For six durum wheat yield trial datasets and the datasets CP and SP, the Savitzky–Golay filter parameter tuning was conducted using the PLSR and rrBLUP prediction models to obtain the combination yielding the highest prediction ability based on the FES. **C** Three breeding scenarios were conducted in this study to assess the use of phenomic prediction in durum wheat breeding programs. Note that in all three breeding scenarios, the environment specifies a location-year combination. In scenario 1, near-infrared spectra (NIRS) and trait data of the training set (TS, e.g. late generation yield trial) and the NIRS of the prediction set (PS, e.g., early generation observation row), which contain each a different sets of genotypes, are obtained within the same environment. Based on the NIRS of the new genotypes, the phenomic estimated performance (PEP) of the new genotypes is predicted. In scenario 2, NIRS and trait data of the TS are obtained in one environment, while the NIRS of the PS are obtained in a new environment aiming to predict the phenotype performance of the same genotypes there, without testing them in large yield trial plots. In scenario 3, series genotype BLUEs_NIRS_ and series genotype BLUEs_trait_ are used as TS. A central environment, in which different (new) genotypes compared to the TS are grown, is used to obtain NIRS. Based on the obtained NIRS, the PEP across different environments is predicted. Between the PEP and the series genotype BLUEs_trait_, the Pearson's correlation is calculated, to evaluate the ability of that environment to predict the phenotype performance over the series of environments, as trait data are not evaluated in the parallel environments due to lacking seed in early generations of the breeding program

We performed feature engineering to improve the prediction ability by testing different kinds of pretreated data in eight different feature engineering scenarios (FES) for phenomic prediction (Table 1, Fig. 1A). For centering (subtraction of column mean) and scaling (division by the columns standard deviation), the *R*-function ‘scale’ was used. Accordingly, the subsequent use of the word ‘scaling’ always implies both: centering and scaling. To evaluate the prediction ability, we conducted a fivefold CV with random assignments of genotypes to folds, replicated 1000 times and determined the CV prediction ability for each FES for the dataset ‘CP and SP combined’. The CP and SP datasets are connected by in total 20 overlapping genotypes. Therefore, the ‘CP and SP combined’ dataset was compiled by calculating BLUEs (Eq. 1) or means across environments resulting for each trait and genotype in a BLUEs (genotype BLUEs_trait_) or a mean (genotype mean_trait_) as well as for each wavelength and genotype in a BLUEs (genotype BLUEs_NIRS_) or mean (genotype mean_NIRS_). The prediction ability was calculated as the Pearson’s correlation coefficient between the predicted genotype values $\left( {\hat{y}} \right)$ and depending on the FES, the genotype BLUEs_trait_ or genotype mean_trait_ $\left( y \right)$ of the respective target trait $\left( {r = cor\left( {\hat{y}, y} \right)} \right)$.

**Table 1:** Feature engineering scenario (FES) overview. Different FESs including scaling (centering and scaling), the calculations of best linear unbiased estimates (BLUEs) or the calculations of arithmetic means (means) and the application of the Savitzky–Golay filter (SG) were compared

Feature engineering scenario	NIRS data				Trait data	
	SG	BLUEs	Mean	Scaling	BLUEs	Mean
FES 1			x			x
FES 2	x		x			x
FES 3			x	x		x
FES 4	x		x	x		x
FES 5		x			x	
FES 6	x	x			x	
FES 7		x		x	x	
FES 8	x	x		x	x

In addition, two prediction models, namely partial least squares regression (PLSR) and ridge regression best linear unbiased prediction (rrBLUP), were used to determine and to compare their prediction ability. rrBLUP, often used in genomic prediction and recently applied in phenomic prediction (Zhu et al. 2022, 2021; Brault et al. 2022; Weiß et al. 2022; Rincent et al. 2018; Dallinger et al. 2023), keeps all wavelengths in the model and shrinks their estimated effects by a constant factor (Whittaker et al. 2000). To apply rrBLUP for phenomic prediction, we used the R package ‘rrBLUP’ (Endelman 2011).

Partial least squares regression, which is classically used in chemometrics (Ferrio et al. 2004, Nagel-Held et al. 2023, Nagel-Held et al. 2022), is reducing the predictor matrix to PLS factors in order to condense the information. Therefore, we used the *R* package ‘pls’ (Mevik and Wehrens 2007) with 12 components. The optimal number of components was chosen based on the root mean-squared error prediction curve using the permutation strategy as implemented in the ‘pls’ *R* package (Mevik and Wehrens 2007). Therefore, the dataset ‘CP and SP combined’ was used. Since GY and PC are major breeding goals in durum wheat breeding and the data availability was highest, we used these two traits for feature engineering, parameter tuning and to analyze the ability of phenomic prediction in this study. The FES yielding the highest prediction ability was used for the subsequent evaluation of breeding scenarios.

In parallel, the best parameter combination of the Savitzky–Golay filter for the NIRS preprocessing was determined within the FES 8 (Table 1) ‘Savitzky–Golay filtered genotype BLUEs_NIRS_ scaled and genotype BLUEs_trait_’ for the dataset ‘CP and SP combined’, the two traits (GY, PC) and the two prediction models (rrBLUP, PLSR). Note that ‘Savitzky–Golay filtered genotype BLUEs_NIRS_ scaled and genotype BLUEs_trait_’ means here, that the NIRS were first Savitzky–Golay filtered, next a BLUE per genotype and wavelength (genotype BLUEs_NIRS_) was calculated and finally, the genotype BLUEs_NIRS_ were centered and scaled. Accordingly, for each genotype and trait, a BLUE (genotype BLUEs_trait_) was calculated.

Therefore, a grid search parameter tuning with iterations over predefined combinations of parameters (polynomial order: 2, 3, 4; derivative order: 1, 2; window size: 29, 39, 49, 59, 69, 79, 89) was performed. The CV prediction ability within each trait and prediction model for each parameter tuning combination was obtained using fivefold CV with random assignment of genotypes to folds, replicated 1,000 times. For each trait and prediction model, the best combination was chosen based on the highest CV prediction ability. In the case of multiple Savitzky–Golay filter parameter combinations yielding the same best CV prediction ability, the less complex combination with lower parameter level height was chosen based on the principle of parsimony. Subsequently, for each prediction model and trait, the best Savitzky–Golay filter combination was used for the other FESs as well as for the breeding scenarios. Moreover, for the six durum wheat yield trials and the datasets CP and SP, the Savitzky–Golay filter combination yielding the highest prediction ability was determined based on the FES 8 (Fig. 1B, Table 1).

### Breeding scenarios

Different use cases exist for breeders to implement phenomic prediction in a breeding program. To evaluate the potential of phenomic prediction in a breeding program, we determined its prediction ability in three different breeding scenarios (Fig. 1C) for two traits (GY, PC). Therefore, we chose the best FES (FES 8, Fig. 3), the best prediction model (rrBLUP) and the best Savitzky–Golay filter parameter combination (Table 3, Table S3) among the tested features and parameters. Our first breeding scenario (Scenario 1) reflects a situation in the early stages of a breeding program, when just enough seed for observation rows but not enough seed to conduct yield trials is available. Here, data of check varieties or genotypes of advanced generations grown in the same environment as the prediction set were used for model training. For the genotypes in early generations (new genotypes, in observation rows), only the NIRS are obtained and trait performance (e.g., GY) is predicted. To validate Scenario 1, within all single environments, a fivefold CV with random assignment of the genotypes to folds, replicated 1000 times, was performed.

In our second breeding scenario (Scenario 2), the situation of early- and mid-generational testing within a wheat breeding program is reflected. In such situations, usually just enough seed is available for a yield trial in one single environment and in all other environments, only ‘miniplots’ just as big to deliver enough seed for the near-infrared spectroscopy can be grown. Hence, the NIRS and trait data obtained in the single environment yield trial (one environment) can be used for model training, while the phenotype performance in the other environments, where no complete yield trial could be performed due to the limited amount of available seed, can be predicted, based on the NIRS obtained in these environments.

Scenario 3 reflects the typical breeding scenario, particularly in the early generations of a breeding program (e.g., F4 generation), when there is only a limited amount of seed available and a breeder is interested in the performance of a genotype across a series of environments.

At this stage, it is impossible to evaluate the performance of a genotype in yield trials in multiple environments, but there is enough seed for yield trials at a single environment. Available spectra and trait data from previous years are used as training set (e.g., ‘CP and SP combined’). A central environment, in which new genotypes compared to the training set are grown, is used to obtain NIRS (‘NIRS environment’). These are used to evaluate the phenomic estimated performance (PEP) across different environments, by assessing the Pearson’s correlation between the PEP (obtained in the ‘NIRS environment’) and the genotype BLUEs_trait_ across the different series of environments (SP series, CP series, YT2-2022 series, YT2-2021 series, YT2-2020 series, YT1-2022 series, YT1-2021 series and YT1-2019 series).

### Discriminant analysis of principal components

For the interpretation of prediction abilities obtained by phenomic selection in this paper, a discriminant analysis of principal components (DAPC) is conducted to identify clusters in our high dimensional datasets (Jombart et al. 2010). Here, NIRS from the CP and SP datasets were used to infer clusters and to compare the inferred clusters with a prior information about genotype assignment to different groups, such as origin or growing environment. Overall, we followed the procedure described by Jombart et al. (2010) using the *R* package ‘MASS’ (Venables and Ripley 2003). First, the dimensionality of the data was reduced using principle component analysis (PCA). Next, *k*-means clustering was performed to infer the optimal number of groups using all principle components. Then, we performed a CV to validate the accuracy of the class assignment of the discriminant analysis using different numbers of principle components. Based on the optimal number of principle components, the *k*-means clustering was run again.

## Results

### Phenotypic data analysis

A total of eight datasets, the Central European durum wheat panel (CP), the South European durum wheat panel (SP), durum wheat yield trial 1 (YT1) conducted in 2019, 2021 and 2022 and durum wheat yield trial 2 (YT2) conducted in 2020, 2021 and 2022 with a total of 884 genotypes (Table S1), were analyzed in this study. All genotypes were evaluated for GY (t ha^−1^) and PC (%) at up to six environments. For each of the different datasets, the mean GY across all genotypes and all environments was calculated and ranged from 5.21 (CP) to 9.26 t ha^−1^ (YT1-2019) and the mean PC from 13.27% (YT2-2021) to 15.61% (YT1-2022; Table 2, Table S2). Heritability for GY within the different datasets ranged from 0.37 to 0.82 and for PC from 0.08 to 0.80, indicating low to high heritabilities (Table 2, Table S2). All subsequent tables and figures in this paper focus on GY, while the tables and figures for PC are presented in the supplementary material.

**Table 2:** Summary statistics for grain yield (GY, t ha^−1^) in durum wheat for the data sets CP, SP, YT1-2019, YT1-2021, YT1-2022, YT2-2020, YT2-2021 and YT2-2022

Trait	Data set	No. Loc	Min	Mean	Max	SED	${\sigma }_{G}^{2}$	${\sigma }_{GxL}^{2}$	${\sigma }_{e}^{2}$	${h}^{2}$
GY (t ha^−1^)	CP	3	4.23	5.21	6.13	0.44	0.19	0.36	0.24	0.48
	SP	6	7.15	7.69	8.40	0.29	0.11	0.24	0.18	0.59
	CP & SP combined	9	5.68	6.64	7.60	0.37	0.15	0.29	0.20	0.54
	YT1-2019	2	8.22	9.26	10.61	0.38	0.39	–	0.33	0.82
	YT1-2021	2	5.49	6.67	7.60	0.33	0.18	–	0.19	0.60
	YT1-2022	2	8.08	8.54	9.16	0.33	0.08	–	0.26	0.37
	YT2-2020	2	5.92	6.83	7.70	0.31	0.21	–	0.22	0.77
	YT2-2021	2	6.23	6.97	7.51	0.42	0.17	–	0.32	0.48
	YT2-2022	2	7.74	8.35	8.87	0.36	0.11	–	0.26	0.43

SED mean standard error of a difference across all pairwise comparisons, ${\sigma }_{G}^{2}$ genotype variance, ${\sigma }_{GxL}^{2}$ genotype–by–environment interaction variance, ${\sigma }_{e}^{2}$ error variance, ${h}^{2}$ heritability

### Decomposition of variance components

**Fig. 2:**
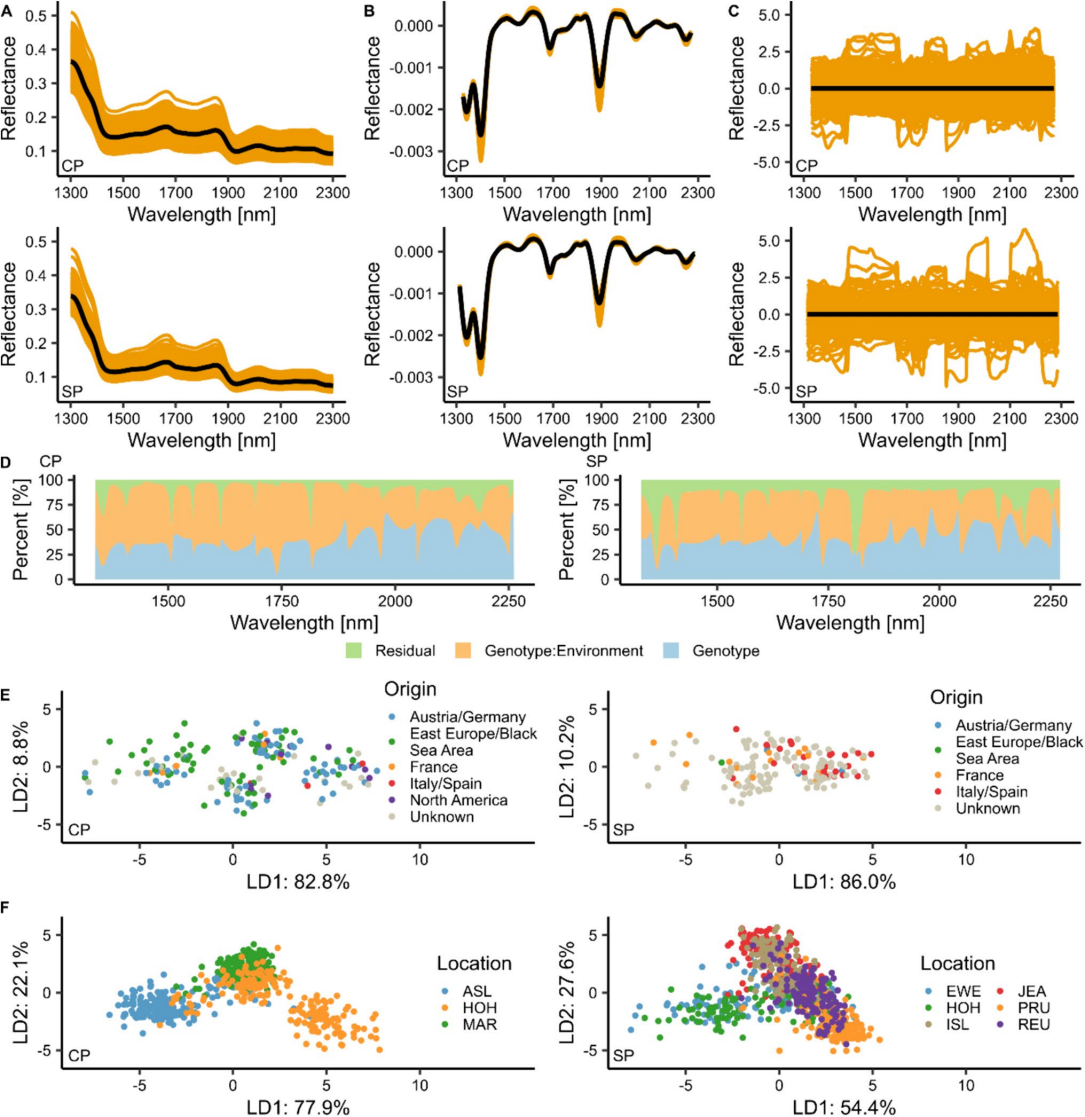
Overview of near-infrared spectra (NIRS), variance components and discriminant analysis of principal components (DAPC) shown separately for the CP and SP datasets. **A** Unprocessed NIRS of the CP and SP dataset, orange lines represent individual genotypes, while the black line indicates the arithmetic genotype mean_NIRS_. **B** Savitzky–Golay filtered genotype BLUEs_NIRS_. **C** Savitzky–Golay filtered as well as centered and scaled genotype BLUEs_NIRS_. **D** Proportion of genotype (blue), genotype-by-environment interaction (orange) and residual (green) variance of each wavelength along the NIRS of durum wheat grains. **E** The DAPC based on NIRS for CP and for SP, the coloring is based on the origin of the genotypes. The amount of variance explained by the first two linear discriminant (LD) functions is plotted below or next to the corresponding axis. **F** DAPC for CP and SP based on NIRS, with coloring according to the growing environments

Unprocessed NIRS were obtained from harvested, dried seed and varied considerably between the datasets (Fig. 2A). The analysis of variance components of unprocessed NIRS (Fig. 2D) revealed a genotype component for all wavelengths as well as a $G\times E$-interaction variance component, but with varying relative contribution and ratio between the wavelengths. The mean genotype variance across all wavelengths for CP was 40.1% (41.3% for SP) and the mean $G\times E$ -interaction variance across all wavelengths for CP was 50.5% (42.1% for SP). The genotype variance of individual NIRS varied considerably from 4.99% to 67.3% for CP and from 9.33% to 73.7% for SP.

### Unraveling the structure of datasets via discriminant analysis of principal components

In the NIRS-based DAPC for genotype origin (Fig. 2E), no distinct clustering was visible for either CP or SP. In contrast to that, the DAPC investigating the influence of environment (Fig. 2F) showed a clustering for the environment. This finding indicates that NIRS contain besides genotype also $G\times E$ -interaction variance. The DAPC showed that the first two linear discriminant functions explained 90.4% (CP) and 99.9% (SP) of the variance, when clustered for origin, while 100% (CP) and 82.0% (SP) were explained when clustered for growing environment.

### Comparison of prediction models and different feature engineering scenarios

**Fig. 3:**
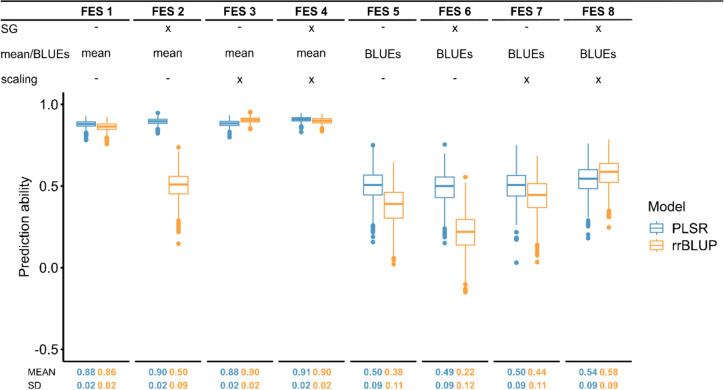
Comparison of the prediction abilities obtained for the eight feature engineering scenarios (FESs) for the trait grain yield. ‘x’ shows that the respective feature (Savitzky–Golay filter (SG), mean or BLUEs and scaling) was included, while ‘–’ shows that the respective feature was not included in the FES. The cross-validated (CV) prediction abilities were obtained by fivefold CV with random assignment of genotypes to folds, replicated 1,000 times for the dataset ‘CP and SP combined’. Two prediction models namely ridge regression best linear unbiased prediction (rrBLUP) and partial least squares regression (PLSR) were used for the predictions. The CV prediction ability (MEAN) and the standard deviation (SD) are plotted below the corresponding FESs

For the dataset ‘CP and SP combined’, we evaluated the CV prediction ability of different prediction models (rrBLUP and PLSR), traits (GY and PC) and FESs using fivefold CV with random assignment of genotypes to folds, replicated 1,000 times (Fig. 3, Fig. S2). For GY, except the FES 2 with the prediction model rrBLUP, all FESs using mean data (mean_NIRS_ and mean_trait_) show higher CV prediction abilities compared to the FESs using BLUEs. All FESs using the prediction model PLSR have in common that the mean prediction abilities are stable within the different data types (mean and BLUEs), even though on different levels. Whereas predictions with the rrBLUP model combined with use of the Savitzky–Golay filter, it seems to depend on the scaling to yield high CV prediction abilities for GY and PC.

### Tuning the Savitzky–Golay filter

For each durum wheat dataset, the best Savitzky–Golay filter combination was determined based on the highest CV prediction ability in a grid search parameter tuning (Table 3). For each model, each trait and each dataset, a different specific combination of parameters resulted in the best CV prediction ability. The range of the highest CV prediction ability resulting from the best parameter combination and the lowest CV prediction ability resulting from the worst parameter combination was different for each model, dataset and trait and had a maximum range of 0.28 (PLSR YT2-2022 GY). For all subsequent breeding scenario predictions and for all subsequent mentioned datasets, the Savitzky–Golay filter parameter combination yielding the highest CV prediction ability in the parameter tuning of the Savitzky–Golay filter was applied (Table 3, Table S3).

**Table 3:** The best Savitzky–Golay filter parameter combinations based on the feature engineering scenario 8 ‘Savitzky–Golay filtered genotype BLUEs_NIRS_ scaled and genotype BLUEs_grain yield_’ for each prediction model, dataset and the trait grain yield resulting from the parameter tuning of the Savitzky–Golay filter (derivative order 1, 2; window size: 29, 39, 49, 59, 69, 79, 89; polynomial order: 2, 3, 4)

Prediction model	Data set	Combinations yielding highest CV prediction ability				Lowest CV prediction ability*
		Polynomial order	Derivative order	Window size	Highest CV prediction ability	
rrBLUP	CP	2	2	79	0.54	0.36
	SP	2	2	59	0.63	0.55
	CP and SP combined	2	2	89	0.58	0.41
	YT1-2019	2	2	59	0.74	0.67
	YT1-2021	2	2	69	0.61	0.46
	YT1-2022	4	2	29	0.32	0.13
	YT2-2020	4	2	89	0.76	0.64
	YT2-2021	2	2	29	0.60	0.46
	YT2-2022	4	2	29	0.33	0.23
PLSR	CP	2	2	89	0.51	0.28
	SP	2	1	69	0.59	0.34
	CP and SP combined	2	2	69	0.54	0.41
	YT1-2019	2	1	79	0.72	0.51
	YT1-2021	2	2	89	0.53	0.26
	YT1-2022	4	1	29	0.41	0.15
	YT2-2020	2	1	89	0.73	0.57
	YT2-2021	3	1	29	0.57	0.37
	YT2-2022	4	1	59	0.30	0.02

rrBLUP, ridge regression best linear unbiased prediction; PLSR, partial least squares regression; CP, central European durum wheat panel; SP, South European durum wheat panel; YT, yield test; *obtained in the Savitzky–Golay filter parameter tuning, parameter combination not shown

### Breeding scenarios

**Fig. 4:**
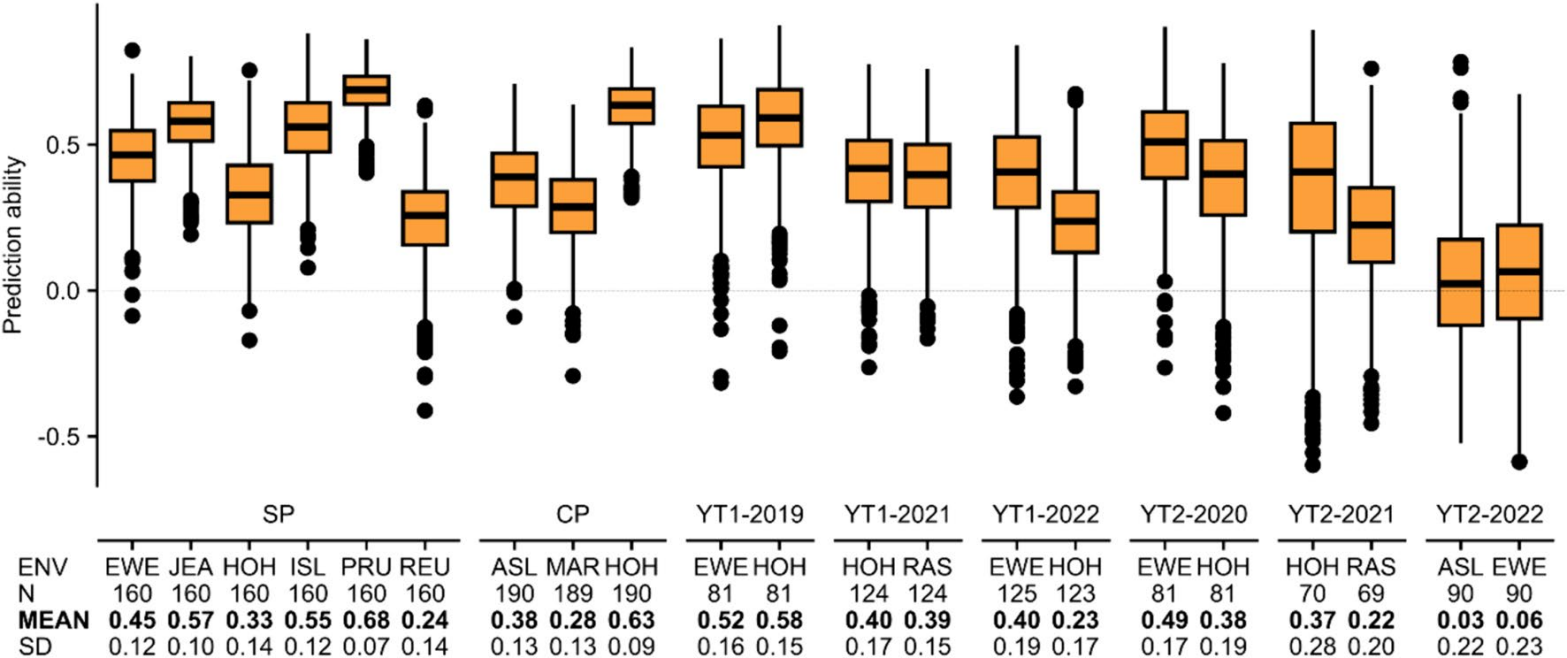
The boxplot shows the prediction abilities for Scenario 1 for the trait grain yield within the 21 environments of the eight different durum wheat datasets obtained in a fivefold CV with 1,000 replicates. Here, the rrBLUP model and the best parameter combination for the Savitzky–Golay filter obtained in the FES 8 ‘Savitzky–Golay filtered genotype BLUEs_NIRS_ scaled and genotype BLUEs_grain yield_’ for each specific durum wheat dataset were used. Below the environments (ENV), the number of tested genotypes per environment (N), the mean CV prediction ability (MEAN) and the standard deviation (SD) is shown

In Scenario 1, we tested the ability to predict new genotypes grown in the same environment as the training set. This resulted for GY in mean CV prediction abilities ranging from 0.03 to 0.68 (Fig. 4) and respective standard deviations ranging from 0.07 to 0.28. For PC, the mean CV prediction abilities ranged from 0.77 to 0.98 and their standard deviations ranged from 0.01 to 0.20 (Fig. S4). The number of lines used for model training ranged from 69 to 190, depending on the environment.

**Fig. 5:**
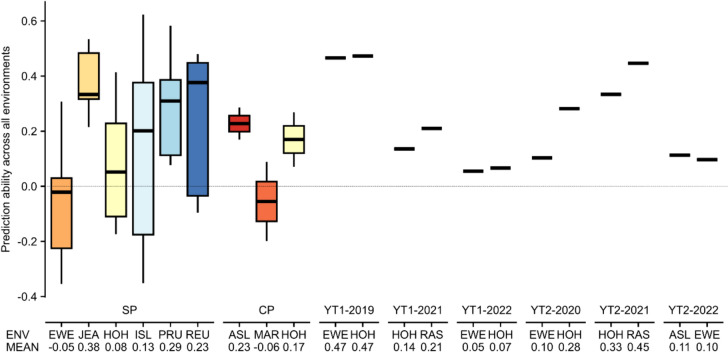
The boxplot shows the prediction abilities obtained for Scenario 2 for the trait grain yield. Here, one environment was used as training environment (ENV) to predict all other environments containing the same genotypes, e.g., when EWE (SP dataset) is used as training environment, the phenotype performances of all other environments belonging to the SP dataset (JEA, HOH, ISL, PRU and REU) are predicted and the five prediction abilities are shown in the boxplot. For the predictions, the rrBLUP model and the best parameter combinations for the Savitzky–Golay filter obtained in the FES 8 ‘Savitzky–Golay filtered genotype BLUEs_NIRS_ scaled & genotype BLUEs_grain yield_’ were used. The arithmetic mean prediction abilities (MEAN) over all predicted environments are shown below the corresponding training environment. Same locations are plotted in the same color

With Scenario 2, we tested the ability to predict observed genotypes in a new environment. This resulted in mean prediction abilities for GY between − 0.06 and 0.47 (Fig. 5) and for PC between 0.78 and 0.97 (Fig. S5). No environment (e.g., HOH in the SP, in the CP, in YT1-2019, YT1-2021, YT1-2022, YT2-2020 or YT2-2021) worked constantly well as a training set (or prediction set; data not shown), i.e., provided consistently good prediction results.

**Fig. 6:**
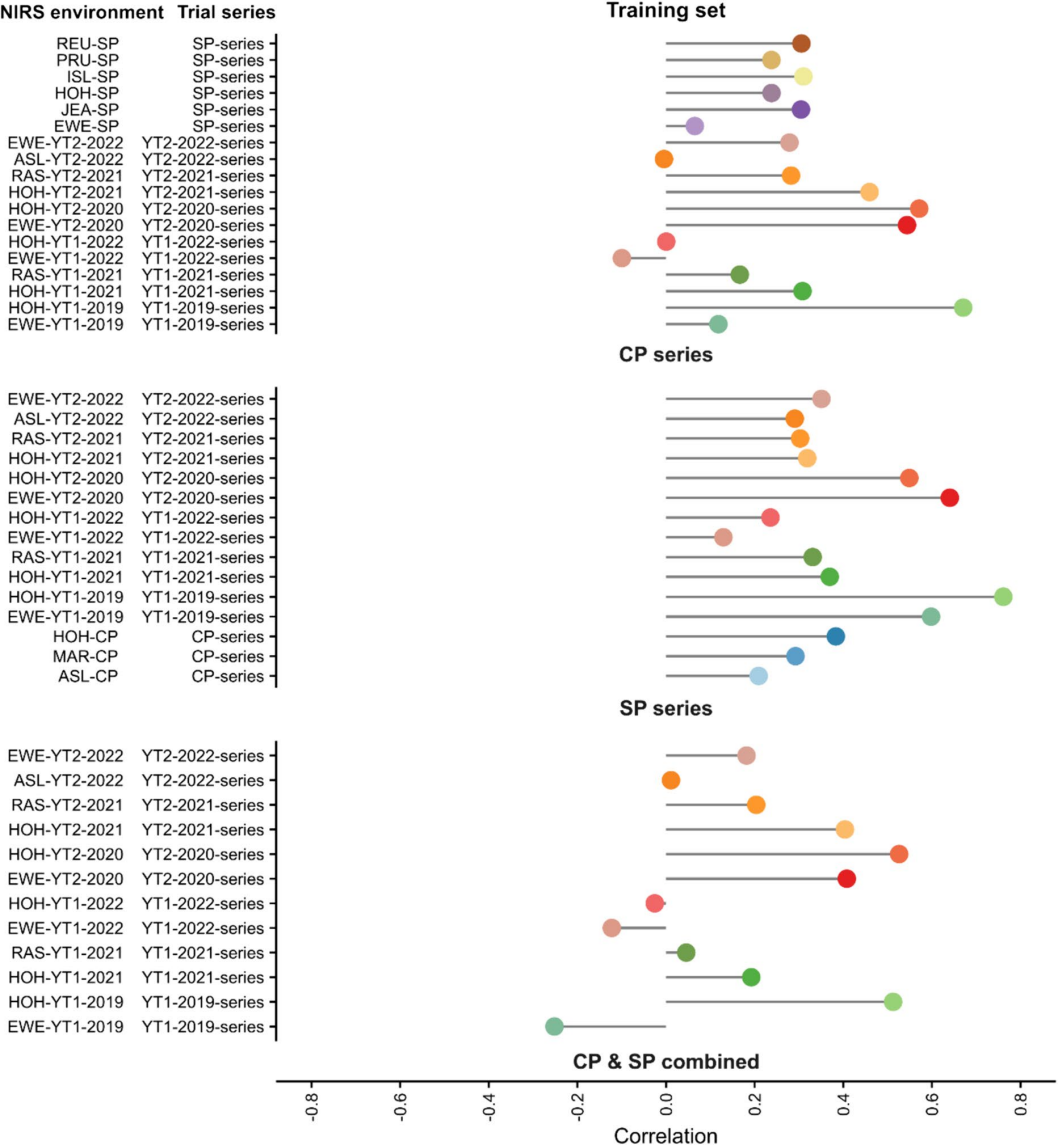
Prediction results of Scenario 3. The different colors represent the different near-infrared spectra (NIRS) environments. Here, an example how to read this plot: genotype BLUEs_NIRS_ and genotype BLUEs_grain yield_ of the CP series (‘Trainingset’, could be historical data for grain yield and NIRS) was used for model training and REU-SP was used as NIRS environment for the new genotypes, which was then used to predict the phenotype performance at a trial series (SP series)

The ability to predict the mean performance of a genotype across a series of test environments based on historical training set data and NIRS from a central environment with the same genotypes as in the environment to be predicted was estimated in Scenario 3. Using the series CP as the training set resulted in a mean prediction ability for GY of 0.29, using the series SP resulted in a prediction ability for GY of 0.36, and using ‘CP and SP combined’ resulted in a mean prediction ability for GY of 0.22. This shows for our case that a larger training set is not improving the prediction ability (Fig. 6, PC Fig. S6).

## Discussion

### Feature engineering and parameter tuning have a major impact on the prediction ability

Before comparing different prediction scenarios in a breeding program, the optimal features and parameter combinations leading to the best prediction abilities need to be identified. Therefore, we investigated in this study the impact of feature engineering, the choice of prediction model and the parameter tuning of the Savitzky–Golay filter on the prediction abilities for two traits (GY and PC) of the durum wheat dataset ‘CP and SP combined’ (Fig. 1).

We evaluated eight feature engineering scenarios (FESs; Table 1, Figs. 1A, 3) with two different prediction models (PSLR and rrBLUP) and two traits (GY, PC; Fig. 3, Fig. S2). For both traits (GY and PC), PLSR performs very stable over the different FESs within the different data types (mean and BLUEs) compared to rrBLUP, which shows a much lower CV prediction ability, when the Savitzky–Golay filter is applied without scaling. Due to the use of the Savitzky–Golay filter, the reflectance values are transformed (Fig. 2A, B), resulting in very small values close to zero. Features with values close to zero exert a pronounced influence on rrBLUP due to the use of L2 penalization, where $\hat{u} = \left( {Z^{\prime } Z + \lambda I} \right)^{ - 1} Z^{\prime } y$, which leads to a shrinkage of the regression coefficients based on the constant $\lambda = \frac{{\hat{\sigma }_{\varepsilon }^{2} }}{{\hat{\sigma }_{u}^{2} }}$ (Henderson 1975), resulting in lower prediction abilities of rrBLUP compared to PLSR, when the Savitzky–Golay filter is applied without scaling. However in PLSR, a dimensionality reduction approach prior to performing ordinary least squares regression (Beebe and Kowalski 1987) is performed. This is increasing the stability of the results even without scaling, thus leading to higher prediction abilities of PLSR compared to rrBLUP, when no scaling was applied. To ensure stable prediction results with rrBLUP, it can be summarized that the Savitzky–Golay filter should be used in combination with scaling, which transforms the spectra and ensures equal variance (Ciurczak et al. 2021) for each spectrum (Fig. 2C).

Moreover, we found that the FES for GY using genotype mean_NIRS_ and genotype mean_trait_ data yield higher CV prediction abilities compared to the scenarios using genotype BLUEs data. The reason for this becomes apparent when considering the correlation between the observed and predicted values e.g., for the FES 1 and the dataset ‘CP and SP combined’ (Fig. S3). For this scenario, the correlation plot shows two clusters of the genotypes belonging to one dataset (CP or SP). The two datasets have different GY levels (Table 2), which are not corrected by the mean calculation but by the mixed model. Therefore, in the cases of the mean data, the prediction abilities are inflated. The mixed model-based BLUEs calculation intends to correct for different experimental design effects (Piepho et al. 2003) and is therefore highly recommended when dealing with phenotype data from different environments. Furthermore, Brault et al. (2022) demonstrated in their study that the use of BLUPs for phenomic prediction resulted in higher prediction abilities compared to scenarios where genotype mean_NIRs_ and genotype mean_trait_ data were used. They also assume an association with the plot coordinate effect or experimental design effects, respectively, for which it is corrected for when BLUPs are calculated.

Beyond that, we conducted for the FES 8 of a Savitzky–Golay filter parameter tuning to fine-tune the Savitzky–Golay filter parameter combinations (Table 3). Within our tested combinations, we obtained an individual best parameter combination for each prediction model, dataset, and trait. The finding that the parameter settings are trait-specific is consistent with Zimmermann and Kohler (2013). Furthermore, these authors found that the optimal window size is restricted to a particular spectral region. Since different spectral regions represent different chemical components that are to some extent trait-related, these specific trait-associated regions should be emphasized in the preprocessing for that trait.

In addition, when comparing the lowest and highest obtained CV prediction ability obtained within our tested combinations, we found that the CV prediction ability could be improved for all datasets and traits, with the highest increase being up to 1500% (0.02–0.3, PLSR YT2-2022 GY). This shows the great potential of the Savitzky–Golay filter parameter tuning, which is therefore highly recommended as a routine for phenomic prediction. Similar results were also found by Robert, Brault, et al. (2022). They confirm that the prediction abilities are influenced by the spectra preprocessing and suggest to test different filters before conducting the deeper analysis. However, fine-tuning the Savitzky–Golay filter is time consuming especially when taking the experimental design into account. For this study, a total of 756,000 BLUE computations were necessary. The computations can be done sequentially in about 5 1/2 days of plain computation time with a laptop Intel(R) Core(TM) i5-10310U CPU @ 1.70 GHz 2.21 GHz 16GB RAM. When accessing a compute cluster, the computation time can be greatly reduced by parallelization. In addition, more sophisticated algorithms such as simulated annealing (Kirkpatrick et al. 1983) could be used to identify a potentially better set of parameter combinations at or near the global optimum. Further research is needed to validate these results on additional datasets using these and other preprocessing approaches.

### NIRS environment has a major impact on the prediction ability

The FES 8 using the rrBLUP model resulted in cross-validated prediction abilities for GY of 0.54 (0.46) for CP, 0.63 (0.51) for SP, while for PC, it resulted in 0.88 (0.46) for CP and 0.93 (0.40) for SP (Table 3, Table S3). In parentheses are the genomic prediction results of Rapp et al. (2018), who used genomic marker data and rrBLUP as prediction model to predict GY and PC within the CP and SP dataset. For comparability, it should be noted that due to the limited availability of NIRS data, only a subset of the environments used for the genomic prediction by Rapp et al. (2018) was also used for phenomic prediction in our study. Even though the genomic prediction results are not fully comparable due to the different training set sizes as well as due to the influence on the phenomic prediction results for PC by using the same spectral data for the phenomic prediction as for the PC determination, it shows that phenomic prediction abilities are higher compared to the genomic prediction abilities. The finding that phenomic prediction delivers higher prediction abilities compared to genomic prediction motivated us to investigate the potential of phenomic prediction to predict the trait performance in more detail for our durum breeding program.

There are several possible ways to implement phenomic prediction in a (durum wheat) breeding program. To evaluate the potential of phenomic prediction, we focused on three different breeding scenarios (Fig. 1C) that seemed to be highly relevant for our durum wheat breeding program. For predicting new genotypes grown in the same environment as the training set, e.g., F4-generation observation rows compared to F6- or later generation YTs (Scenario 1), we obtained low-to-moderate prediction abilities of 0.03–0.68 for GY and even higher ones for PC. As the square root of the heritability is an upper limit for the prediction ability (Zhang et al. 2019), prediction abilities larger than ~ 0.5 are promising for early stage selection of genotypes under the Scenario 1 conditions.

In Scenario 2, we predicted the trait performance of the same genotypes as used for model training based on spectra obtained in a new environment, i.e., we need to grow the genotypes in the new environments but only in small plots. The results show prediction abilities for GY in the range of − 0.06 to 0.47 (Fig. 5) and for PC in the range of 0.78–0.97 (Fig. S5), meaning that no training environment is clearly suitable for stable predictions. This is in agreement with Ferrio et al. (2004), who reported challenges when spectra were collected in environments different from those in which the predictions were made. They concluded that calibrations should be made within the same environments as the predictions, which is also consistent with our results for Scenario 1 (prediction of new genotypes grown in the training environment), which showed higher prediction abilities compared to those obtained in Scenario 2.

When interpreting these results, it should be considered that NIRS capture both, the genetic as well as the $G\times E$-interaction variance. In our study, when considering the mean variance across the range of spectra considered in this study, on average 40.1% (50.5%) and 41.3% (42.1%) of the NIRS variance for CP and SP dataset, respectively, can be attributed to the genotype variance. In parentheses, these are the $G\times E$-interaction variance of the NIRS (Fig. 2D). These variance proportions are in agreement with other studies (Rincent et al. 2018; Robert et al. 2022c; Brault et al. 2022) indicating a strong influence of the environment on the prediction abilities when applying phenomic prediction. As NIRS capture both, genotype and environmental effects, it is not surprising that phenomic prediction tends to outperform genomic prediction under certain conditions (Weiß et al. 2022; Zhu et al. 2022).

We further examined the NIRS using a DAPC analysis (Fig. 2E). No population structure could be detected based on the origin of the genotypes, whereas a separation based on marker data was detected by Sieber et al. (2017) for origin. We then used the single environment NIRS to perform the DAPC, and a relatively clear separation of the environments was revealed (Fig. 2F), supporting the results of the variance component decomposition that the NIRS capture a large proportion of the $G\times E$ -interaction. Results from Delwiche et al. (2002) suggest that NIRS are sensitive to environmental conditions during the period of grain development, which must be taken into consideration when using these data.

It is important for breeders to know the performance of their varieties across a wide range of environments within their commercial target region as early as possible. Therefore, in Scenario 3, we tested the prediction ability using sets of historical NIRS and trait data for model training and one environment (central environment) to obtain NIRS of the latest variety candidates for prediction. Based on the NIRS obtained in this central environment, the phenotype of the variety candidates is predicted and the correlation between the predicted phenotype and the series genotype BLUEs_trait_ (calculated across a wide range of environments) is calculated. Please note, that in an applied situation, the new genotypes would not be evaluated in field trials. This scenario is similar to the common use of genomic prediction, where markers are obtained based on plant material grown in one central environment and the performance is predicted based on historical training data. The results show low-to-moderate prediction abilities (Fig. 6, S6).

The mean prediction abilities for GY with 0.29 when using the CP series, 0.36 when using the SP series and 0.22 when using CP and SP combined as training set show that larger training set sizes are not resulting in better mean prediction abilities in our study. This and the finding of negative correlations can be explained by the strong year and environment effect observed in the data and the fact that we did not explicitly account for the $G\times E$ interaction in the phenomic prediction model. Furthermore, the datasets are either not connected with common genotypes (YT datasets) or only with a low number of check genotypes. We consider Scenario 3 a realistic scenario and assumption for applied breeding programs with a limited budget. Explicit modeling of $G\times E$ interaction was beyond the scope of this study but can be considered as beneficial depending on the assumed scenario (Robert et al. 2022c).

When considering the implementation of phenomic prediction based on NIRS in a durum wheat breeding program, we can summarize that the suitability of phenomic prediction depends on the breeding scenario. Especially in situations like in Scenario 1, an implementation of phenomic prediction seems promising for PC and also for GY, a trait with lower heritability. In general, the high prediction abilities obtained for PC in the feature engineering, parameter tuning and the practice scenarios are not unexpected, since we trained the prediction model rrBLUP on the same spectral data as used for the PC determination. Nevertheless, the effectiveness of the feature engineering and parameter tuning could be proven also for this trait.

### Study limitations and further research questions

To perform model selection based on parameter tuning and feature selection, we used fivefold CV repeated 1000 times. In each repetition, 80% of the data were randomly sampled to form the training set and the remaining 20% of the data were used for the test set. Applying this procedure for model selection is in alignment with the findings by Wager (2020) who proofed numerical that CV is suitable for model selection. The use of CV for parameter tuning and model selection is further supported by Wainer and Cawley (2021), which found strong evidence for this by comparing 12 algorithms on 115 empirical datasets. Further Bates et al. (2023) showed that if model selection is not the main interest of using CV, but assessing the prediction error of a model, then nested CV based on simulations should be used, especially when the sample size (n) is < 100. For n > 100 and especially n >  = 400, CV performs similarly to nested CV. In a nested CV, an inner and an outer CV are used. The inner CV is used, for example, to select the features or to tune the parameter combinations that lead to the highest prediction ability within the search space tested, while an outer CV is used to evaluate the performance of the model. It should be noted that nested CV is way more computationally intensive than non-nested CV (Wainer & Cawley 2021). The question arises whether the previously mentioned findings also apply to the preprocessing of NIRS in the context of phenomic. This can be addressed in further research projects.

In addition to that, we would like to stress again that the high prediction abilities observed for PC are not unexpected due to use of the same spectral data for both the prediction and the PC determination. Consequently, further studies should be conducted to reanalyze the ability of phenomic prediction to predict PC in durum wheat based on protein content values obtained from chemical analysis.

Furthermore, the prediction ability of phenomic prediction in durum wheat should be evaluated in further studies, using a larger and more balanced dataset with more genotypes, environments and preferably more overlapping genotypes. Moreover, when considering the breeding scenarios across environments, covariables describing the environment, such as weather data, could improve the prediction ability. In addition to that, as already done for other crops (Robert et al. 2022c), the combination of genomic and phenomic data should be tested to predict GY and PC in durum wheat with higher accuracy.

## Conclusion

In the present study, we investigated the application of phenomic prediction in a durum wheat breeding program, focusing on the evaluation of different scenarios relevant to such a program, the optimization of spectral preprocessing, the feature engineering and the mechanisms underlying phenomic prediction. Our results showed that the optimal Savitzky–Golay filter parameter combinations used to preprocess the spectral data depend on the dataset, the trait and the prediction model and that their optimization is highly beneficial to improve the prediction ability. In addition to that, we demonstrated that instead of arithmetic means for the genotype_NIRS_ and genotype_trait_, linear-mixed model-based adjusted means should be used in order to correct for the experimental design effects, especially when the data are unbalanced. Furthermore, we demonstrated that NIRS capture a large proportion of $G\times E$-interaction variance in the datasets under investigation. Among the breeding scenarios tested, we found the highest prediction abilities when predicting new genotypes based on their spectra obtained in the same environment as the training set data. However, the prediction abilities were rather low and unstable across environments.

### Supplementary Information

Below is the link to the electronic supplementary material.

Supplementary file1 (DOCX 769 KB)

## Data Availability

The data analyzed during this study are not publicly available due to breeding programs privacy but are available from the corresponding author on reasonable request.
