# Supplementary material for: Feature engineering and parameter tuning: improving phenomic prediction ability in multi-environmental durum wheat breeding trials
Source: Theor Appl Genet. 2024 Jul 22;137(8):188. doi: 10.1007/s00122-024-04695-w (PMC11263437; doi:10.1007/s00122-024-04695-w)
Supplement: Supplementary file 1 — Supplementary file1 (DOCX 769 KB) [file 122_2024_4695_MOESM1_ESM.docx]

**Supplementary Material**

**Feature Engineering and Parameter Tuning - Improving Phenomic Prediction Ability in Multi-Environmental Durum Wheat Breeding Trials**

Carina Meyenberg^1^, Vincent Braun^1^, Carl Friedrich Horst Longin^1^, Patrick Thorwarth^1^

^1^ State Plant Breeding Institute, University of Hohenheim, Fruwirthstr. 21, 70599 Stuttgart, Germany

Corresponding author: Patrick Thorwarth, patrick.thorwarth@uni-hohenheim.de

**Email addresses:**

CM: c.meyenberg@uni-hohenheim.de

VB: vincent.braun@uni-hohenheim.de

CFHL: friedrich.longin@uni-hohenheim.de

PT: patrick.thorwarth@uni-hohenheim.de

**ORCID IDs:**

CM: 0009-0008-3456-3553

VB: 0009-0004-7076-8899

CFHL: 0000-0002-0737-1651

PT: 0000-0003-4456-2358


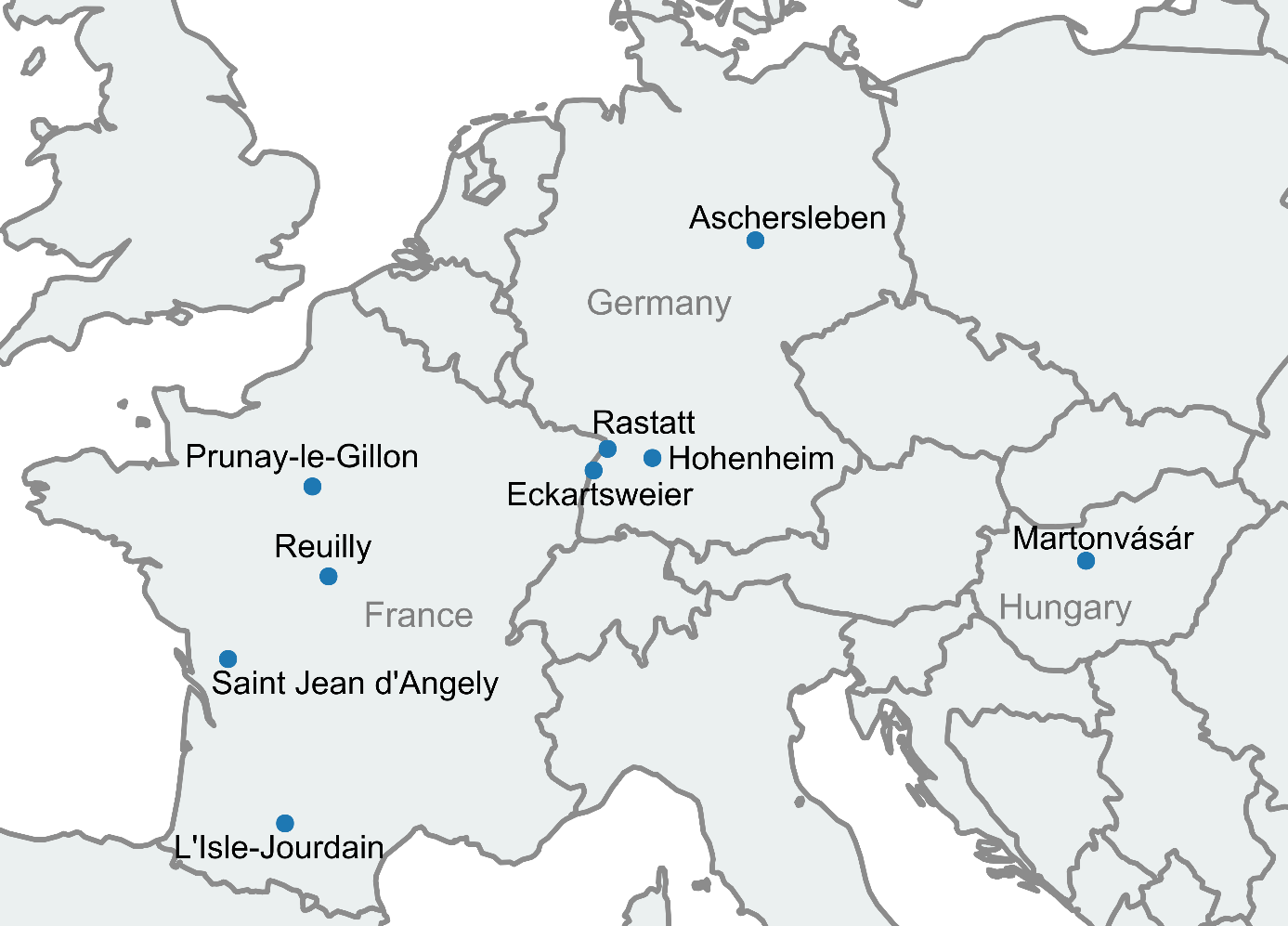


*Figure S 1 Overview map over the nine trial locations used within CP, SP, YT1 and YT2, located in France, Germany and Hungary.*

*Table S 1 Overview of the four data sets used in this study. Trait shows the arithmetic mean for grain yield (GY) and protein content (PC) for a respective environment (location-year combination).*

| **Data- set** | **Year of harvest** | **Location** | **Number of tested genotypes** |  | **Trait** | |
| --- | --- | --- | --- | --- | --- | --- |
|  |  |  |  |  | **GY**  (t ha^-1^) | **PC**  (%) |
| CP | 2016 | Aschersleben | 190 |  | 68.17 | 14.21 |
|  |  | Martonvásár | 189 |  | 30.19 | 16.11 |
|  |  | Hohenheim | 190 |  | 56.30 | 13.47 |
| SP | 2015 | Saint Jean d’Angély | 160 |  | 68.99 | 15.34 |
|  |  | Eckartsweier | 160 |  | 96.85 | 13.33 |
|  |  | Hohenheim | 160 |  | 87.97 | 12.69 |
|  |  | L’Isle-Joudain | 160 |  | 50.09 | 16.36 |
|  |  | Prunay-le-Gillon | 160 |  | 85.08 | 14.66 |
|  |  | Reuilly | 160 |  | 73.69 | 15.71 |
| YT1 | 2019 | Eckartsweier | 81 |  | 90.38 | 14.23 |
|  |  | Hohenheim | 81 |  | 95.90 | 16.35 |
|  | 2021 | Hohenheim | 124 |  | 71.37 | 14.56 |
|  |  | Rastatt | 124 |  | 62.63 | 13.37 |
|  | 2022 | Eckartsweier | 125 |  | 88.54 | 16.07 |
|  |  | Hohenheim | 123 |  | 82.02 | 15.08 |
| YT2 | 2020 | Eckartsweier | 81 |  | 71.93 | 13.54 |
|  |  | Hohenheim | 81 |  | 67.01 | 15.25 |
|  | 2021 | Hohenheim | 70 |  | 78.07 | 14.69 |
|  |  | Rastatt | 69 |  | 62.61 | 12.06 |
|  | 2022 | Aschersleben | 90 |  | 74.89 | 14.43 |
|  |  | Eckartsweier | 90 |  | 90.99 | 15.84 |

*Table S 2 Summary statistics for protein content (PC) in durum wheat for the data sets CP, SP, YT1-2019, YT1-2021, YT1-2022, YT2-2020, YT2-2021, YT2-2022.*

| trait | | data set | No. Loc | Min | Mean | Max | SED | $\sigma_{G}^{2}$ | $\sigma_{GxL}^{2}$ | $\sigma_{e}^{2}$ | $h^{2}$ |
| --- | --- | --- | --- | --- | --- | --- | --- | --- | --- | --- | --- |
| PC (%) | | CP | 3 | 13.58 | 14.55 | 16.19 | 0.45 | 0.22 | 0.31 | 0.24 | 0.55 |
|  |  |  |  |  |  |  |  |  |  |  |  |
|  |  | SP | 6 | 13.45 | 14.72 | 16.28 | 0.40 | 0.32 | 0.20 | 0.39 | 0.75 |
|  |  | CP & SP combined | 9 | 13.31 | 14.67 | 16.46 | 0.45 | 0.30 | 0.28 | 0.31 | 0.66 |
|  |  |  |  |  |  |  |  |  |  |  |  |
|  |  | YT1-2019 | 2 | 13.82 | 14.88 | 16.67 | 0.43 | 0.27 | - | 0.27 | 0.65 |
|  |  | YT1-2021 | 2 | 12.93 | 13.87 | 14.97 | 0.38 | 0.23 | - | 0.16 | 0.69 |
|  |  | YT1-2022 | 2 | 15.51 | 15.61 | 15.72 | 0.19 | 0.02 | - | 0.30 | 0.08 |
|  |  |  |  |  |  |  |  |  |  |  |  |
|  |  | YT2-2020 | 2 | 13.19 | 14.52 | 15.94 | 0.48 | 0.57 | - | 0.27 | 0.80 |
|  |  | YT2-2021 | 2 | 12.33 | 13.27 | 14.59 | 0.36 | 0.23 | - | 0.15 | 0.72 |
|  |  | YT2-2022 | 2 | 14.11 | 15.09 | 15.77 | 0.45 | 0.23 | - | 0.22 | 0.55 |
|  | SED mean standard error of a difference across all pairwise comparisons, $\sigma_{G}^{2}$ genotype variance, $\sigma_{GxL}^{2}$ genotype-by-environment interaction variance, $\sigma_{e}^{2}$ error variance, $h^{2}$ heritability | | | | | | | | | | |


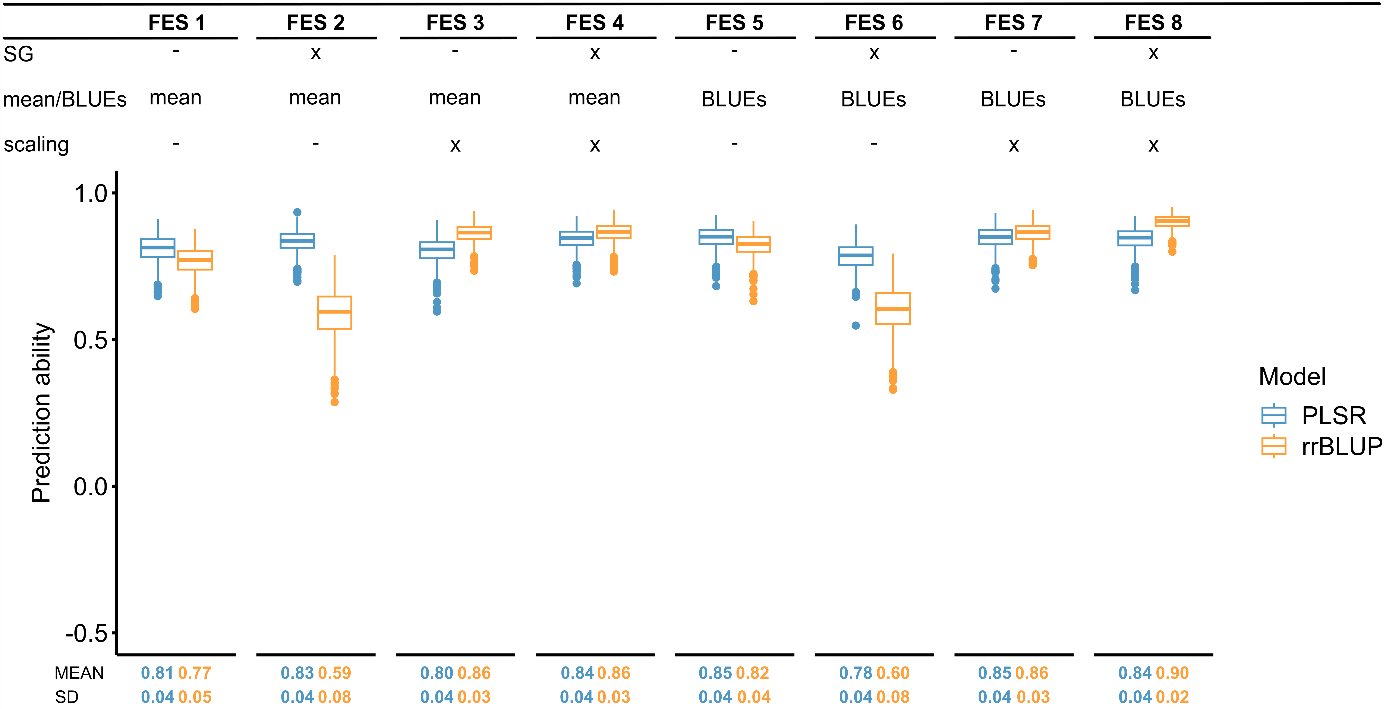


*Figure S 2 Comparison of the prediction abilities obtained for different feature engineering scenarios (FESs) for the trait protein content. ‘x’ shows that the respective feature (Savitzky-Golay filter (SG), mean or BLUEs and scaling) was included, while ‘–‘ shows that the respective feature was not included in the FES. The cross-validated (CV) prediction abilities were obtained by fivefold CV with random assignment of genotypes to folds, replicated 1,000 times for the data set ‘CP & SP combined’. Two prediction models namely ridge regression best linear unbiased prediction (rrBLUP) and partial least squares regression (PLSR) were used for the predictions. The CV prediction ability (MEAN) and the standard deviation (SD) are plotted below the corresponding FESs.*


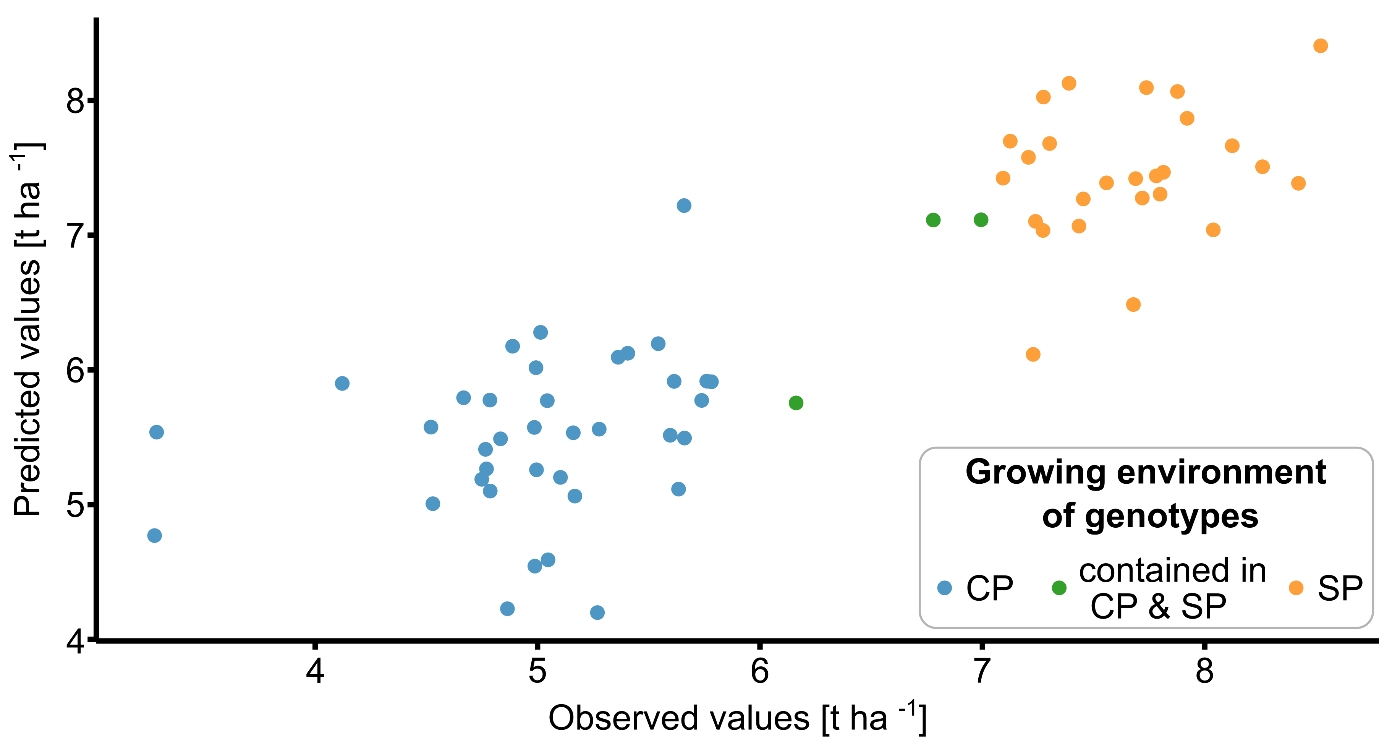


*Figure S 3 Correlation between observed values and predicted values for one cross-validation replication of the feature engineering scenario 1 ‘genotype mean_NIRS_ & genotype mean_Grain Yield_’ based on the data set ‘CP & SP combined’ and the trait grain yield. The colors indicate the origin of genotypes from the different data sets.*

*Table S 3 The best Savitzky-Golay filter parameter combinations based on the feature engineering scenario 8 ‘Savitzky-Golay filtered genotype BLUEs_NIRS_ scaled & genotype BLUEs_protein content_’ for each prediction model, data set and the trait protein content resulting from the parameter tuning of the Savitzky-Golay filter (derivative order 1, 2; window size: 29, 39, 49, 59, 69, 79, 89; polynomial order: 2, 3, 4).*

|  |  | combination yielding highest  CV prediction ability | | | |  |
| --- | --- | --- | --- | --- | --- | --- |
| prediction model | data set | polynomial order | derivative  order | window size | highest CV prediction ability | lowest CV  prediction  ability* |
| rrBLUP | CP | 2 | 2 | 29 | 0.88 | 0.81 |
|  | SP | 2 | 1 | 29 | 0.93 | 0.89 |
|  | CP & SP combined | 4 | 2 | 29 | 0.93 | 0.87 |
|  | YT1-2019 | 2 | 1 | 29 | 0.98 | 0.90 |
|  | YT1-2021 | 2 | 1 | 29 | 0.97 | 0.89 |
|  | YT1-2022 | 2 | 1 | 29 | 0.95 | 0.79 |
|  | YT2-2020 | 2 | 2 | 69 | 0.95 | 0.88 |
|  | YT2-2021 | 2 | 2 | 69 | 0.92 | 0.77 |
|  | YT2-2022 | 3 | 1 | 29 | 0.93 | 0.81 |
| PLSR | CP | 4 | 2 | 29 | 0.88 | 0.81 |
|  | SP | 2 | 1 | 29 | 0.93 | 0.88 |
|  | CP & SP combined | 4 | 2 | 29 | 0.92 | 0.84 |
|  | YT1-2019 | 2 | 2 | 69 | 0.98 | 0.83 |
|  | YT1-2021 | 2 | 1 | 29 | 0.96 | 0.88 |
|  | YT1-2022 | 3 | 1 | 49 | 0.94 | 0.77 |
|  | YT2-2020 | 2 | 1 | 39 | 0.94 | 0.88 |
|  | YT2-2021 | 2 | 2 | 69 | 0.91 | 0.79 |
|  | YT2-2022 | 3 | 1 | 29 | 0.93 | 0.83 |
| rrBLUP: ridge regression best linear unbiased prediction, PLSR: partial least squares regression, CP: central European durum wheat panel, SP: south European durum wheat panel, YT: yield test; *obtained in the Savitzky-Golay filter parameter tuning, parameter combination not shown | | | | | | |

*
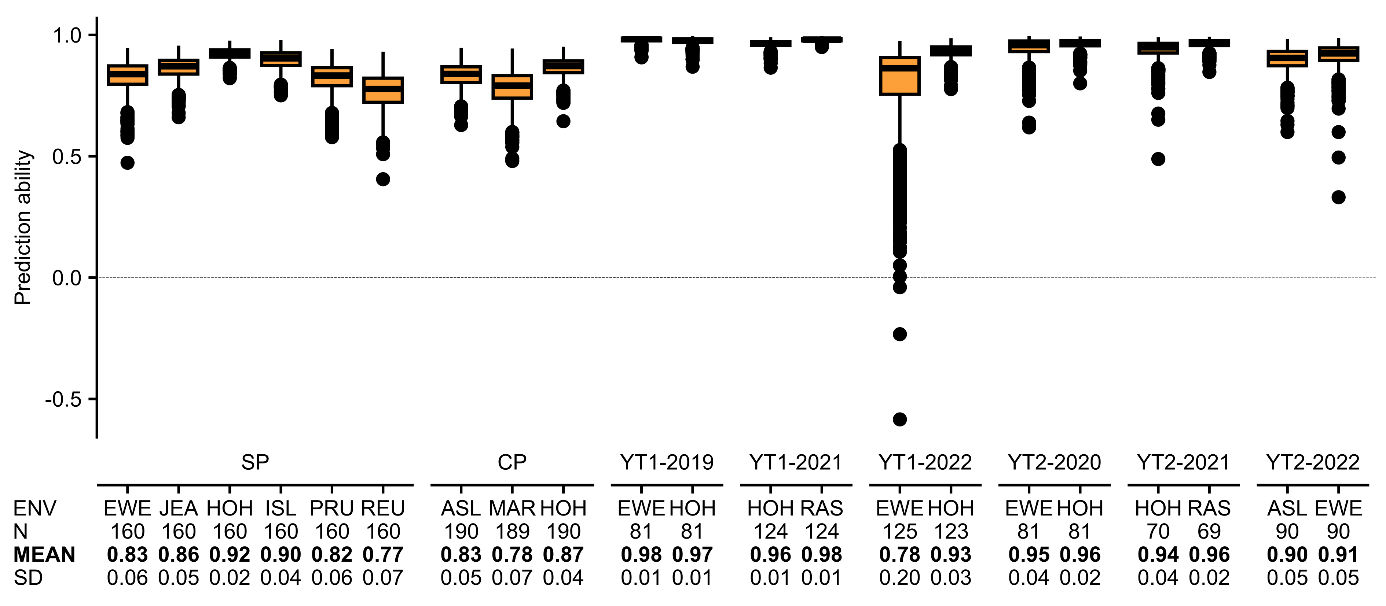
*

*Figure S 4 The boxplot shows the prediction abilities for Scenario 1 for the trait protein content within the 21 environments of the eight different durum wheat data sets obtained in a fivefold CV with 1,000 replicates. Here, the rrBLUP model and the best parameter combination for the Savitzky-Golay filter obtained in the FES 8 ‘Savitzky-Golay filtered genotype BLUEs_NIRS_ scaled & genotype BLUEs_protein content_’ for each specific durum wheat data set were used. Below the environments (ENV), the number of tested genotypes per environment (N), the mean CV prediction ability (MEAN) and the standard deviation (SD) are shown.*

*
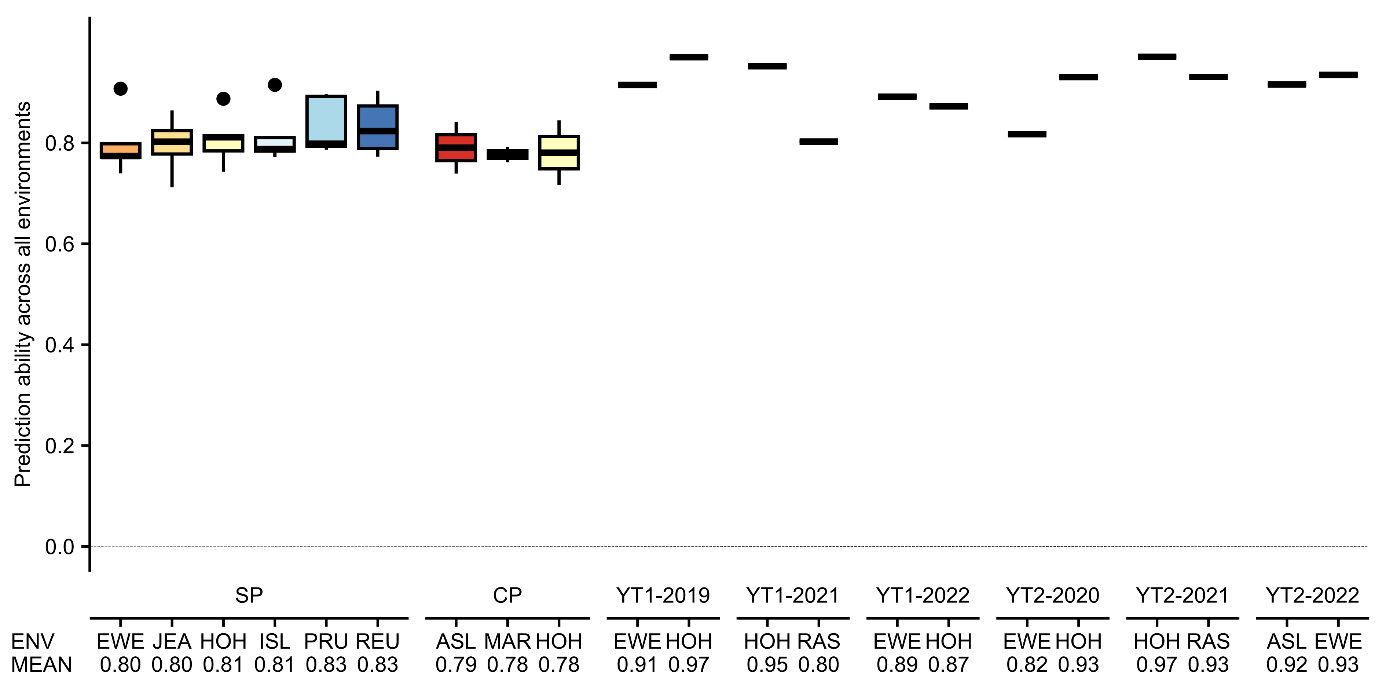
*

*Figure S 5 The boxplot shows the prediction abilities obtained for Scenario 2 for the protein content. Here, one environment was used as training environment (ENV) to predict all other environments containing the same genotypes, e.g. when EWE (SP data set) is used as training environment, the phenotype performances of all other environments belonging to the SP data set (JEA, HOH, ISL, PRU and REU) are predicted and the five prediction abilities are shown in the boxplot. For the predictions the rrBLUP model and the best parameter combinations for the Savitzky-Golay filter obtained in the FES 8 ‘Savitzky-Golay filtered genotype BLUEs_NIRS_ scaled & genotype BLUEs_protein content_’ were used. The arithmetic mean prediction abilities (MEAN) over all predicted environments are shown below the corresponding training environment. Same locations are plotted in the same color.*


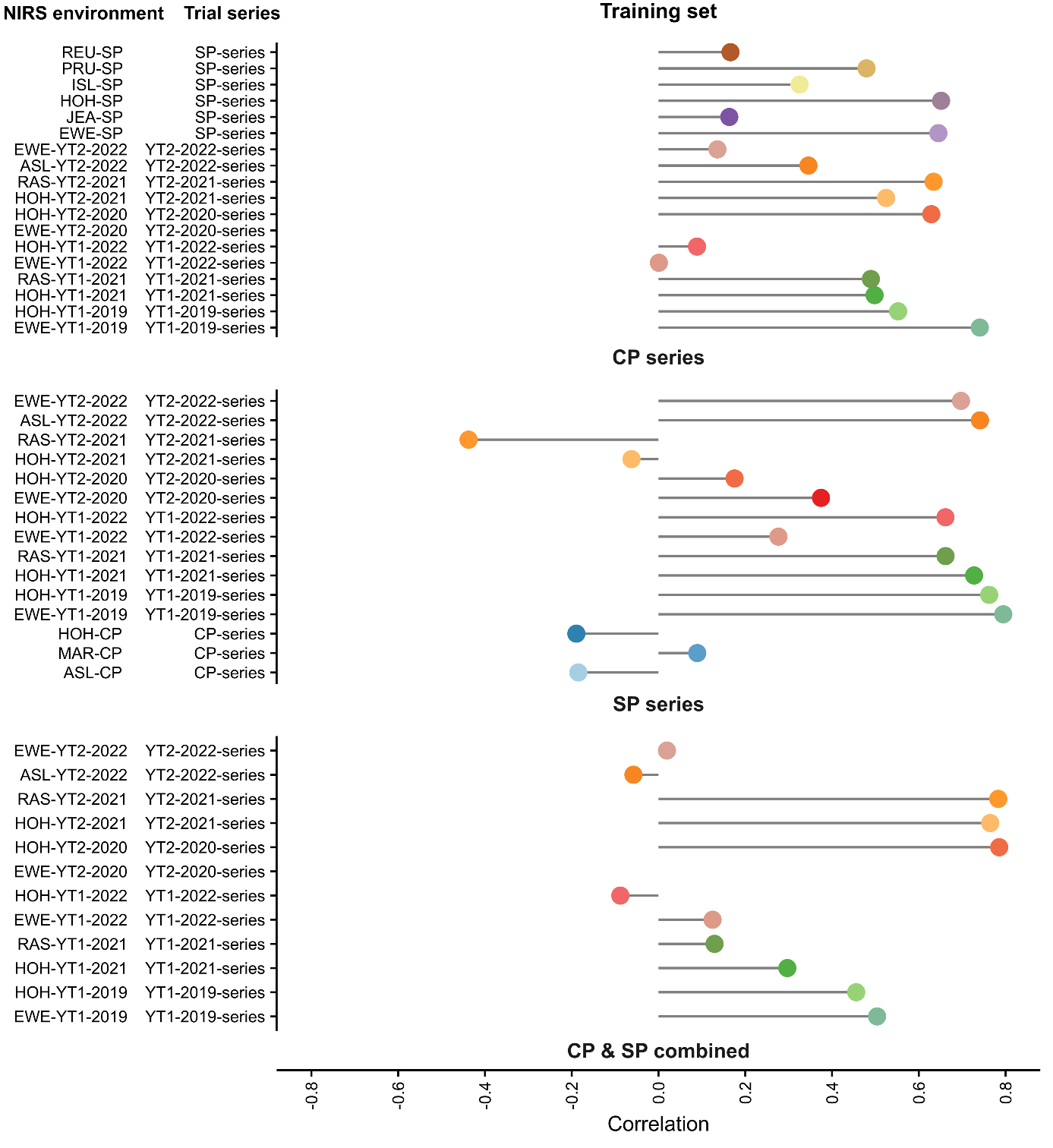


*Figure S 6 Prediction results of Scenario 3. The different colors represent the different near-infrared spectra (NIRS) environments. Here an example how to read this plot: genotype BLUES_NIRS_ and genotype BLUEs_protein content_ of the CP series (‘Trainingset’, could be historical data for protein content and NIRS) was used for model training and REU-SP was used as NIRS environment for the new genotypes, which was then used to predict the phenotype performance at a trial series (SP-series).*
